# The Screen for Child Anxiety-Related Emotional Disorders Is Sensitive but Not Specific in Identifying Anxiety in Children with High-Functioning Autism Spectrum Disorder: A Pilot Comparison to the Achenbach System of Empirically Based Assessment Scales

**DOI:** 10.3389/fpsyt.2017.00138

**Published:** 2017-08-02

**Authors:** W. David Lohr, Katherine Daniels, Tim Wiemken, P. Gail Williams, Robert R. Kelley, Grace Kuravackel, Lonnie Sears

**Affiliations:** ^1^Department of Pediatrics, University of Louisville School of Medicine, Louisville, KY, United States; ^2^School of Psychology, Spalding University, Louisville, KY, United States; ^3^University of Louisville School of Public Health and Information Sciences, Louisville, KY, United States; ^4^St. Mary’s College of Maryland, St. Mary’s City, MD, United States

**Keywords:** autism, anxiety, screening instruments, self-report, assessment

## Abstract

Validated brief screening instruments are needed to improve the detection of anxiety disorders in autism spectrum disorder (ASD). The Screen for Child Anxiety-Related Emotional Disorders (SCARED), a 41-item parent- and self-reported scale measuring anxiety, was compared to the Achenbach System of Empirically Based Assessment (ASEBA) scales. One hundred participants with a clinical diagnosis of high-functioning ASD, aged 8–18 years, and their parents completed the above scales. We hypothesized that the SCARED would be useful in screening for anxiety and its results for total scores of anxiety would converge with ASEBA syndrome scales for anxiety and internalizing disorders. Significant correlations were shown between the SCARED and the Child Behavior Checklist (CBCL) and Youth Self-Report (YSR) across a broad spectrum of scales. The CBCL syndrome scale for anxious/depressed showed the highest correlation and predicted anxiety scores on the SCARED. While many of the YSR scales significantly correlated with child ratings of anxiety, none of the scales predicted the SCARED child scores. Differences in self and parent reports suggest that parents interpret externalizing behaviors as signs of anxiety in ASD, whereas youth may describe internalized symptoms as anxiety. Females were more likely to self-report anxiety than males. Results support the use of the SCARED as a screening tool for anxiety in high-functioning ASD, but it should be supplemented with other tools to increase the specificity of its results.

## Introduction

Psychiatric comorbidity represents an important source of increased impairment and dysfunction in autism spectrum disorders (ASD). In a recent study using a structured interview, 72% of children with high-functioning autism had at least one other psychiatric disorder, with the most common diagnoses being specific phobia (44%), obsessive–compulsive disorder (37%), ADHD (30.6%), and major depression (10.1%) (1).

Anxiety disorders are recognized as a leading source of additional impairment in ASD symptoms with estimates of comorbid anxiety symptoms as high as 84% (2). In addition, a meta-analysis showed 39.6% of young people with ASD had at least one comorbid DSM-IV anxiety disorder, with specific phobia the most common (3). Compared to typical children with anxiety disorders, those children with comorbid ASD and anxiety have more total anxiety, social anxiety, and panic (4). The increased morbidity, dysfunctional behavior, and exacerbation of ASD symptoms caused by anxiety disorders are important to detect because they can provide targets for clinical intervention (5). For example, cognitive behavioral therapy has been shown to reduce anxiety symptoms, improve disruptive and repetitive behaviors, and improve social function (6, 7).

Sensitive and specific measures of anxiety in a brief clinical format are needed to improve the clinical ability to diagnose and treat anxiety in ASD. Despite the high prevalence, it is often difficult for clinicians to make the diagnosis of anxiety disorders in children with ASD. First, it is not clear whether anxiety disorder and ASD represent true independent conditions or if anxiety symptoms are a part of the core definition of ASD (8). Symptoms of anxiety may present as non-specific behaviors or as an increase in repetitive behaviors (2). Second, self-reports may be inconclusive in youth with ASD due to problems with emotional insight or ability to accurately describe and report one’s emotions (9). Many youth with ASD struggle to understand and describe their inner experiences and emotional language in detail (10, 11). Relying on parent report may also be problematic since parents and children with ASD in general do not show good correlation in their reports of anxiety (12). Third, youth with ASD with aberrant behaviors and co-occurring psychiatric conditions are more likely to be treated with psychotropic medication polypharmacy (13). Since youth with ASD also have high rates of medical comorbidity such as seizures (14), efforts to increase the accuracy of comorbid psychiatric disorders can allow for more targeted and safer treatments. A clinically useful and accurate instrument measuring anxiety in ASD is an important step toward improving the ability to identify problems and measure outcomes in this population (15).

Several studies have reviewed the strengths and weaknesses of brief measures of anxiety in ASD (8, 16, 17). Concerns have been raised regarding the use of self-reported measures of anxiety in ASD since they often have low correlation with structured interview data from parents (9). Parents may report observable behavior such as rituals, restlessness, insomnia, and discomfort in social situations as anxiety, while items requiring direct expression by the patient are less frequently endorsed. Those children with the highest levels of anxiety showed a great deal of externalizing behavior and irritability (18). Parent and child reports using the Spence Children’s Anxiety Scale-revised, the Revised Children’s Anxiety and Depression Scale (RCADS), and the Screen for Child Anxiety-Related Emotional Disorders (SCARED), have shown acceptable structure and reliability (19), but parent–child correlation using measures of anxiety are generally only low to moderate (16).

Reliable and valid assessment of anxiety in children can be aided by adding teacher reports to the clinical picture (20). Teachers have shown a high level of inter-rater agreement when assessing severity of autism (21). Teachers and parents perceive externalized symptoms of attention-deficit/hyperactivity disorder in a similar manner when asked to rate children with ASD but have less agreement when rating internalized symptoms such as anxiety (22, 23). Finally, teachers tend to report lower rates of internalizing psychiatric symptoms in students with ASD (24).

The Screen for Child Anxiety-Related Emotional Disorders is a well-established screening instrument for anxiety in youth (25). The SCARED is a 41-item parent- and child-report scale that is free for download, quick to use, and provides a summary score of anxiety symptoms. High internal consistency and correlation with structured interview was shown. It also shows promising results in its application to children with ASD. Both European and American versions of the SCARED show good internal reliability and convergent validity with structured clinical interviews in ASD. It represents scores of anxiety in a normal distribution with a mild positive skew suggesting a higher cutoff may be necessary (26). Parents, in general, report more anxiety than children using the scale but at least moderate correlation was shown (27). Some adjustment of the cut off scores may be needed to optimize sensitivity and specificity (17).

The Achenbach System of Empirically Based Assessment (ASEBA) family of instruments is well validated to study global psychopathology in children (28) and has been used to discriminate those with ASD from other diagnoses (29). Several ASEBA measures that allow for a comprehensive approach to identifying functioning in youth include: the Child Behavior Checklist (CBCL) 6–18 years, Youth Self-Report (YSR) 6–18 years, and Teacher Report Form (TRF) 6–18 years. The ASEBA measures are well suited for initial evaluations and research applications, as they require data entry and software to provide results. While the CBCL was not designed to screen for ASD, it has been shown to be useful in identifying emotional problems in children with intellectual disability and ASD (30). The instrument supports an internalizing–externalizing factor structure in ASD and the comorbid diagnosis of anxiety disorder (31). It has gained support in measuring anxiety and depression in ASD and serves as a useful comparison given its sound psychometric properties and dimensional characteristics (32). Problems with lower specificity have been explained by some overlap with symptoms of ASD (33).

It is important to continue to compare self-report instruments such as the SCARED with other validated tools in measuring anxiety in ASD. Our study is the first to report the relationship between the ASEBA instruments and the SCARED in ASD and offers a dimensional framework for evaluating the relationship of anxiety disorders to autism and how parent and child reports of anxiety differ in this population. Our study focused on children with ASD without estimated intellectual disability because of the likelihood of greater success completing self-report measures requiring the ability to read and answer written questions.

The objectives of our study were first to examine correlations of the SCARED-child and -parent forms in a population of youth with ASD without intellectual disability to the ASEBA instruments of global psychopathology (CBCL, YSR, and TRF). Second, we aimed to understand the reported behaviors from parents, youth, and teachers that would predict ratings of anxiety. Specifically, we wished to evaluate how parents and children’s reports of anxiety in ASD agree and the extent to which teacher reports of behaviors relate to parent and child reports of anxiety. This aim will add to the current literature on the differences between parent and child reports of anxiety in ASD. Finally, as a secondary aim, the effect of gender on parent and child reports of anxiety was measured.

Our main hypothesis was that the SCARED would be useful in screening for anxiety with convergence between total scores of anxiety and ASEBA syndrome scales for anxiety and internalizing disorders. Also, we hypothesized the triangulation between parent, child, and teacher, ASEBA reports of global psychopathology would give a deeper understanding of what behaviors and symptoms are being reported as anxiety in subjects with ASD. It was also expected that, similar to populations of youth without ASD, female subjects with ASD would report more symptoms of anxiety.

## Materials and Methods

### Participants

100 patients (88 males, 12 females) aged 8–18 years (mean age 12.9 years) were recruited from a university child psychiatry clinic, a tertiary regional diagnostic developmental clinic, and a university autism center. Referrals were generated from posted notices and from treating clinicians in the clinics. Inclusion criteria required subjects have an established clinical diagnosis of ASD without estimated intellectual disability. In 64 subjects, the diagnosis of ASD was made by psychological testing including the use of the ADOS. In 36 subjects, a clinical diagnosis of ASD was made by trained clinical professionals (psychologists, physicians with autism expertise) according to DSM-IV and DSM-5 criteria. The primary author reviewed all diagnoses. The sample identified participants as having diagnoses of autism (*n* = 47), Asperger’s disorder (*n* = 40), and pervasive developmental disorder-not otherwise specified (*n* = 13). The labels of Asperger’s disorder, and pervasive developmental disorder, not otherwise specified were retained if made under DSM-IV criteria. Estimated intellectual function was determined by review of available cognitive (*n* = 28) or educational testing data (*n* = 26), parent report of academic and adaptive functioning (*n* = 30), or interview with subject to determine levels of academic function and verbal expression (*n* = 16). Comorbid diagnoses were obtained from review of the subject’s medical records. All subjects and families completed informed consent as approved by the University of Louisville Institutional Review Board.

### Materials

The Screen for Child Anxiety-Related Emotional Disorders is a 41-item child- and parent- rated measure of anxiety for youth aged 4–18 years. It has shown acceptable reliability, validity, sensitivity, and specificity and has been studied in many populations, including those with ASD (25, 27, 34).

The Achenbach System of Empirically Based Assessment is designed to assess a broad range of emotional and behavioral syndromes. The CBCL 6–18, Youth Self Report 11–18, and TRF 6–18 years all feature population norm-referenced scales from the viewpoint of different informants (28). The scales measure emotional and behavioral problems on a three-point Likert Scale and feature internalizing/externalizing dimensional scales, DSM-oriented scales, and eight syndrome scales. The ASEBA is widely used in medical and educational settings and has been studied across numerous cultures and settings. The CBCL has demonstrated acceptable psychometric properties in youth with ASD (31).

### Procedures

Guardians and subjects were introduced to the study and provided informed consent and assent prior to participating. One hundred participants and parents completed the 41-item SCARED-child and -parent forms and paper copies of the CBCL and YSR (if the subjects were at least 11 years old). Subjects were judged by inclusion criteria to be capable of reading and completing the questionnaires without assistance. While parents were available to provide clarification if needed, the subjects were encouraged to read and complete the instruments without support. Also, guardians were asked to have a teacher of their choosing complete a TRF (44 out of 100 were returned by mail or guardian). If necessary, reminder phone calls were made to guardians at 6 weeks requesting return of the TRF. ASEBA raw scores scales were entered into the ASEBA data management system to provide competence, broadband, syndrome, and DSM-oriented scales. Raw scores for each scale were converted to norm-referenced *T*-scores that allow indication as either clinical or borderline significance.

Pearson’s correlation was used to evaluate the correlations between SCARED-child and -parent scores and the *T*-scores for broadband, syndrome, and DSM-oriented scales from the CBCL, TRF, and YSR. Linear regression models were also used to identify the scales within the CBCL, TRF, or YSR that best predicted child and parent SCARED scores. Linear regression models were used to assess associations between SCARED-child and -parent scores and the effect of the child’s age, sex, race, and level of school were each evaluated by multivariable linear regression.

## Results

The average age of the subjects was 13.00 years, median age 13.25 years (range 8.08–18.67 years), and average grade of the subjects was 7.17. The majority of the subjects, *n* = 88, were males and 86% of parents self-classified as Caucasian. 87 out of 100 subjects had comorbid psychiatric diagnoses (see Table 1) and anxiety disorders and ADHD were the most common comorbid condition (see Table 2). Teacher reports were obtained for *N* = 44 subjects and YSR were obtained for those subjects aged 11–18 years (*N* = 72 subjects). Both child and parent SCARED scores were significantly correlated with many scales of the CBCL and YSR as described below.

**Table 1:** The number of subjects with comorbid psychiatric diagnoses.

Number of comorbid psychiatric diagnoses	Number of subjects
Zero	*N* = 13
One	*N* = 22
Two	*N* = 34
Three	*N* = 28
Four	*N* = 2
Five	*N* = 1

	Total *N* = 100

**Table 2:** Numbers of subjects with each psychiatric diagnosis.

Anxiety disorder/generalized anxiety disorder	*N* = 70
ADHD	*N* = 66
Obsessive–compulsive disorder	*N* = 15
Major depression	*N* = 12
Impulse control disorder NOS	*N* = 8
Mood disorder NOS	*N* = 6
Posttraumatic stress disorder	*N* = 5
Oppositional defiant disorder	*N* = 4

	Total *N* = 100 subjects

### SCARED-Parent and -Child Correlations to the CBCL

**Figure 1:**
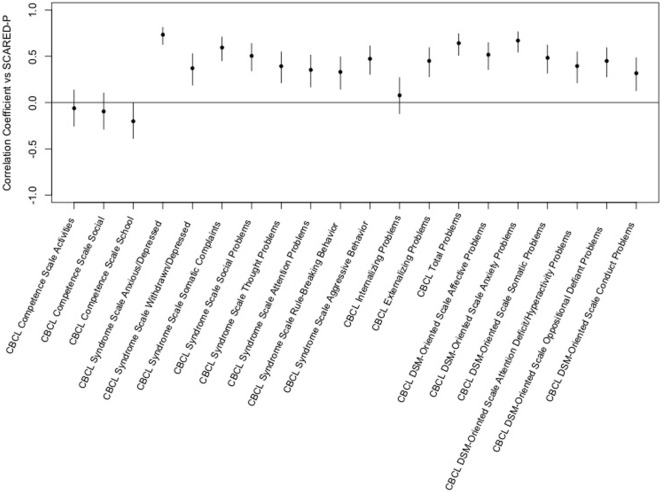
Correlation of the Screen for Child Anxiety-Related Emotional Disorders (SCARED)-parent vs. child behavior checklist (CBCL) *T*-scores. This figure shows the correlation coefficients and 95% confidence intervals for association of each variable within the CBCL with the sums of parent ratings of anxiety on the SCARED. Significance is present when the 95% confidence interval does not cross the horizontal line at 0. Dots/lines above the line represent positive correlation and those below the horizontal line are negative correlation.

The Screen for Child Anxiety-Related Emotional Disorders parent scores were positively correlated with CBCL syndrome scales for anxious/depressed, withdrawn/depressed, somatic complaints, social problems, thought problems, attention problems, rule-breaking behavior, and aggressive behavior. Parental ratings of anxiety correlated with externalizing and total problems but not internalizing problems on the CBCL. Significant correlations were also seen between SCARED-parent scores and all of the CBCL DSM-oriented scales but the highest correlation were for anxiety problems. Overall, the highest correlation was between parent ratings of anxiety and the CBCL syndrome scale of anxious/depressed (see Figure 1).

**Figure 2:**
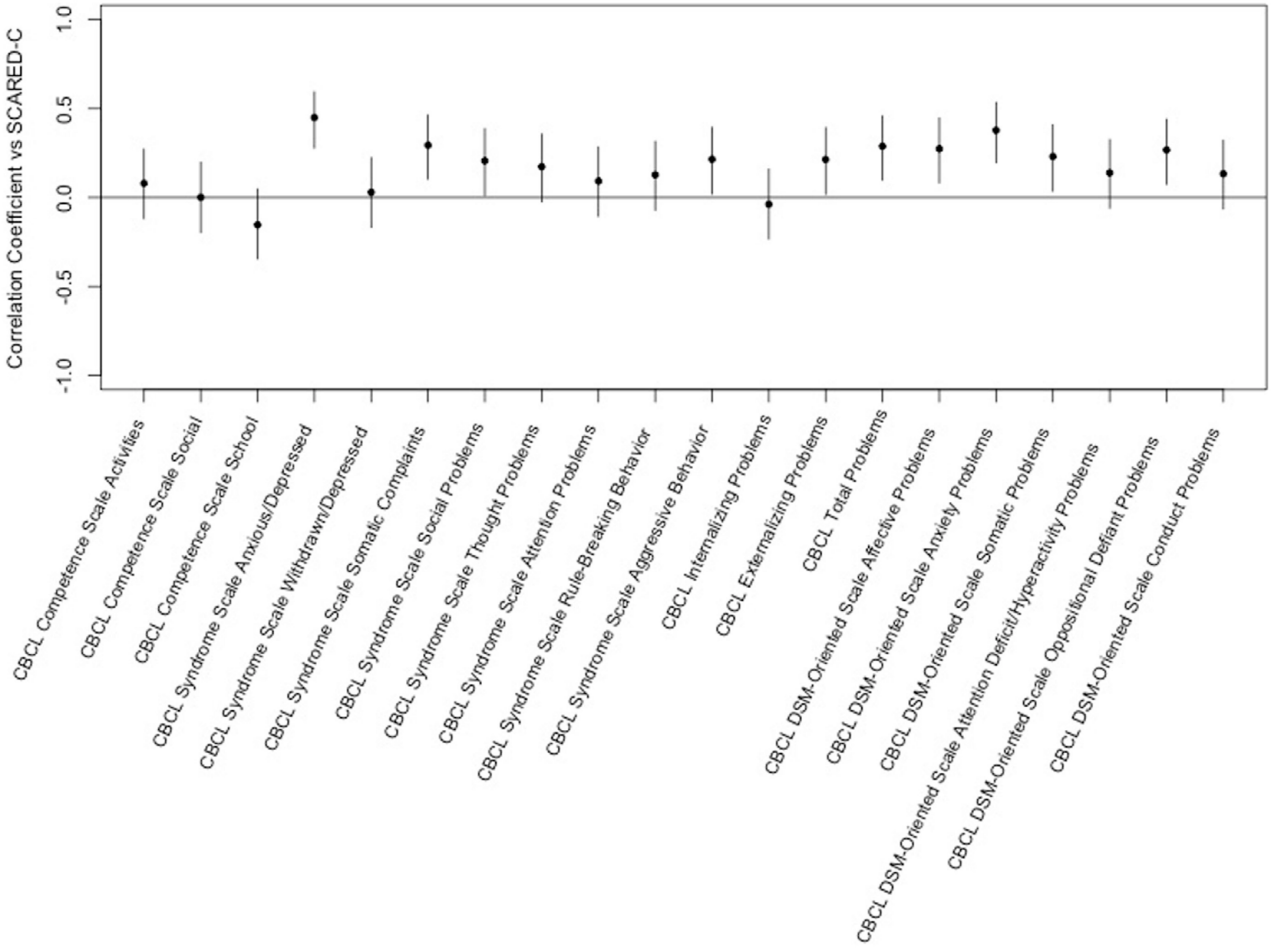
Correlation of the Screen for Child Anxiety-Related Emotional Disorders (SCARED)-child vs. child behavior checklist (CBCL) *T*-scores. This figure shows the correlation coefficients and 95% confidence intervals for association of each variable within the CBCL with the sums of child ratings of anxiety on the SCARED. Significance is present when the 95% confidence interval does not cross the horizontal line at 0. Dots/lines above the line represent positive correlation and those below the horizontal line are negative correlation.

The Screen for Child Anxiety-Related Emotional Disorders-child scores were also positively correlated with several scales of the CBCL although the magnitudes of the correlations were less. Significant correlations were seen for CBCL syndrome scales for anxious/depressed, somatic complaints, social problems and aggressive behavior and for externalizing and total problems but not internalizing problems. Significant correlations were seen between SCARED-child scores and CBCL DSM-oriented scales for affective problems, anxiety problems, somatic problems, and oppositional defiant problems. The highest correlation was between child ratings of anxiety and the CBCL syndrome scale of anxious/depressed (see Figure 2).

### SCARED-Parent and -Child Correlations to the YSR

**Figure 3:**
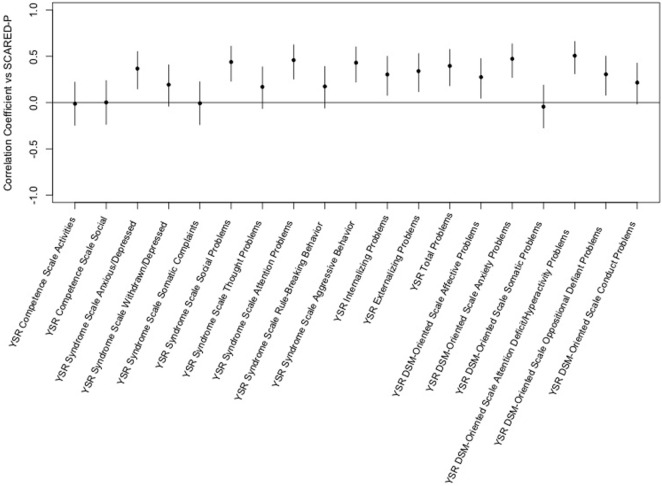
Correlation of the Screen for Child Anxiety-Related Emotional Disorders (SCARED)-parent vs. Youth Self-Report (YSR) *T*-scores. This figure shows the correlation coefficients and 95% confidence intervals for association of each variable within the YSR with the sums of parent ratings of anxiety on the SCARED. Significance is present when the 95% confidence interval does not cross the horizontal line at 0. Dots/lines above the line represent positive correlation and those below the horizontal line are negative correlation.

The Screen for Child Anxiety-Related Emotional Disorders-parent scores were positively correlated with YSR Syndrome Scales for anxious/depressed, social problems, attention problems, and aggressive behavior. Significant correlation was seen for internalizing, externalizing, and total problems and for DSM-oriented scales for affective problems, anxiety problems, attention-deficit/hyperactivity problems, and oppositional defiant problems. Interestingly, the highest degree of correlation was seen between the SCARED-parent and the youth reported scale for attention-deficit/hyperactivity problems (see Figure 3).

**Figure 4:**
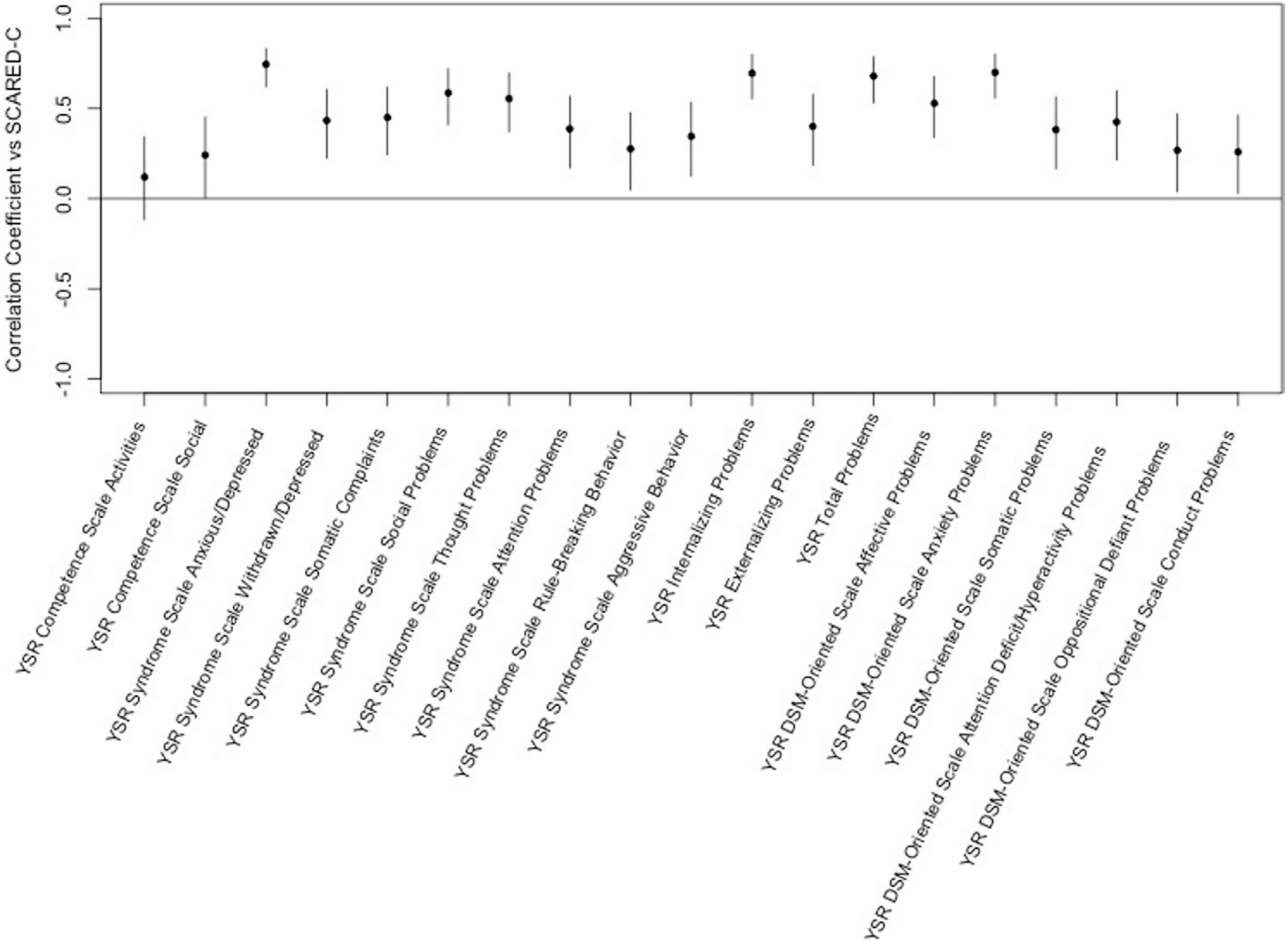
Correlation of the Screen for Child Anxiety-Related Emotional Disorders (SCARED)-child vs. Youth Self-Report (YSR) *T*-scores. This figure shows the correlation coefficients and 95% confidence intervals for association of each variable within the YSR with the sums of child ratings of anxiety on the SCARED. Significance is present when the 95% confidence interval does not cross the horizontal line at 0. Dots/lines above the line represent positive correlation and those below the horizontal line are negative correlation.

The Screen for Child Anxiety-Related Emotional Disorders-child scores significantly correlated with all but one of the YSR scales. The highest correlation was for YSR Syndrome Scale of Anxious/Depressed. The only YSR scale that was not positively correlated with the SCARED-child was the YSR Competence Scale Activities Scale (see Figure 4).

### SCARED-Parent and -Child Correlations to the TRF

Only the TRF Syndrome Scale of Anxious/Depressed significantly correlated to the SCARED-parent scale with a lower magnitude compared to CBCL and YSR. The SCARED-child scale did not significantly correlate with any of the TRF syndrome, broadband, or DSM-oriented scales.

In summary, the highest correlations between SCARED-parent and SCARED-child scores and the CBCL was for the syndrome anxious/depressed scale. The same finding held for the SCARED-child and YSR. Both parent and child ratings of anxiety severity were significantly correlated with CBCL reports of externalizing behavior but not internalizing behavior. Youth scores of anxiety on the SCARED were more correlated with internalizing symptoms on the YSR, but this was not seen for SCARED-parent reports. Few correlations were found between the SCARED scores and TRF.

### Linear Regression Models

The CBCL syndrome scale for anxious/depressed significantly predicted both SCARED-child (beta coefficient = 0.88 and *p*-value = 0.008) and -parent (beta coefficient = 0.48 and *p*-value = 0.013) scores.

Higher CBCL DSM-scale ratings for oppositional defiant problems (beta coefficient = 0.46, *p*-value = 0.027) and lower CBCL competence scale scores for activities (beta coefficient = −0.33, *p*-value = 0.019) predicted ratings of anxiety symptoms on the SCARED-parent.

Youth Self-Report syndrome scales for aggressive behavior (beta coefficient = 1.2, *p*-value = 0.04) and DSM-oriented scale for affective problems (beta coefficient = 0.82, *p*-value = 0.04) and anxiety problems (beta coefficient = 1.1, *p*-value = 0.002) positively predicted SCARED-parent scores. YSR syndrome scale scores for anxious/depressed (beta coefficient = −1.0, *p*-value = 0.049) and somatic complaints (beta coefficient = −1.1, *p*-value = 0.017) negatively predicted SCARED-parent scores. Although many of the YSR scales were significantly correlated with child ratings of anxiety, none of the YSR scales predicted the SCARED-child scores. None of the TRF scales significantly predicted SCARED-parent scores but the TRF DSM-oriented scale for conduct problems negatively predicted scared-child scores (beta coefficient = −4.4, *p*-value = 0.014).

### Parent–Child Correlation for Ratings of Anxiety

Medium correlation between parent and child SCARED scores was found (*r* = 0.43, with a *p*-value of <0.001). The 95% confidence interval for this correlation coefficient was 0.26–0.58. Linear regression resulted in a beta coefficient of 0.50, SE of 0.10, a *t*-statistic of 4.8 and a *p*-value of <0.001. This suggests that for each point increase in the SCARED-parent score, the SCARED-child score increased by 0.5 points. Yet, only about 18% of the variation of the SCARED-child form was explained by the SCARED-parent score (adjusted *R* squared = 18.1%).

Other potentially confounding variables between parent and child ratings of anxiety were explored. Similar results to the unadjusted model were found when the effect of child’s age, race, and grade in school (grade school vs. middle school or high school), were examined. The only demographic variable that was significantly associated with the child SCARED score was sex (beta coefficient = 12.2, SE = 4.5, *t*-statistic 2.7, *p*-value = 0.008). This suggests that the SCARED-child score is higher (12 points higher) if the child is female vs. male (adjusted *R* squared = 24%), indicating this model explains more of the variation in the child SCARED scores than the model not including these other variables.

## Discussion

The main objective of this study was to review a brief, clinically valid instrument assessing anxiety symptoms in the ASD population. To accomplish this, our study examined the correlations of the SCARED-child and -parent forms in a population of youth with ASD without intellectual disability to the ASEBA instruments of global psychopathology (CBCL, YSR, and TRF). Our results support the use of the SCARED-child and -parent instrument in ASD to measure anxiety. The SCARED instruments are readily available at no cost, can be administered and scored within a few minutes, and could be easily used to provide a sensitive tool to improve the detection of anxiety in ASD.

The SCARED-child and SCARED-parent forms show significant correlation with the CBCL and YSR across a broad spectrum of scales. The CBCL and YSR syndrome scales for anxious/depressed had the highest correlation and significantly predicted both child and parent SCARED scores indicating some convergence between the instruments. However, since the SCARED significantly correlates with many of the CBCL and YSR scales, the association is non-specific and further exploration is needed. This is the first known report of comparing the SCARED-child and -parent to the well-validated and dimensional ASEBA instruments of global psychopathology and illustrates important features of how anxiety in ASD is recognized by children, parents, and teachers.

Anxiety in ASD can be viewed broadly in terms of internalizing and externalizing dimensions as measured by the CBCL. Parents may see externalizing behavioral symptoms as signs of anxiety in children with ASD. The CBCL broadband scale of externalizing problems significantly associated with both child and parent reports of anxiety and the highest degree of correlation between the YSR and the SCARED-parent was for the DSM-oriented scale for attention-deficit/hyperactivity problems. Higher parent ratings for oppositional defiant problems and lower parent scores for activities predicted how anxious parents felt their children were. Parent ratings of anxiety in their children with ASD were also predicted by the youth’s own rating of aggressive behavior. Parents may consider disruptive behavior, resistance to compliance, and decreased involvement in social activities as signs of anxiety in their children with ASD. These findings differ from parent reports of anxiety using the SCARED in non-ASD populations, which show significant correlations with CBCL internalizing factors (35, 36).

Youth with ASD may more likely interpret internalized symptoms as anxiety. Youth scores of anxiety on the SCARED were more correlated with internalizing symptoms on the YSR and SCARED-child scores were predicted by self-reports of affective and anxiety problems. However, youth scores of anxiety on the SCARED had broad range of significant correlations across many of the ASEBA scales indicating non-specificity. Seemingly higher self-reports of both internalizing and externalizing symptoms are associated with higher reports of anxiety on the SCARED. Moderate agreement between SCARED-child and SCARED-parent reports of anxiety was seen. SCARED-parent scores had significant correlation with several YSR syndrome and DSM-oriented scales including those targeting anxiety.

Teacher reports of anxiety *via* the TRF syndrome scale of anxious/depressed correlate to parent, but not child reports of anxiety. There was no significant correlation between TRF scales and child reports of anxiety. For teachers, increased awareness of disruptive and negative behaviors predicted lower child scores of anxiety and they may have a lack of recognition of internalized symptoms in children with ASD. In the classroom, teacher reports of conduct problems identify a group of ASD students with low self-report of anxiety.

There may be gender differences in parent and child reports of anxiety in children with ASD as parents and female patients with ASD agree more closely in their reports of anxiety. Previous studies have shown effect of gender on expression of anxiety in ASD. Females with autism have been shown to have higher internalizing symptoms and depression than boys (37, 38). Females with ASD show greater increases in ratings of depression and anxiety over time (32). Our findings also suggest females with ASD may self-report more anxiety than males, although the small number of females in our sample, *N* = 12, limits our ability to draw conclusions.

Our study raises other questions about how anxiety in subjects with ASD is measured *via* instruments. The correlations between the SCARED-child and -parent scales and CBCL are broad-based, non-specific, and the prediction of results of anxiety scores by YSR syndrome scales was in the unexpected negative direction. Although many of the YSR scales were significantly correlated with child ratings of anxiety, none of the YSR scales predicted the SCARED-child scores. Interestingly, the correlations were non-specifically significant on all syndrome scales and DSM-oriented scales between SCARED-parent and the CBCL and between the SCARED-child and YSR. That is, the more anxious a parent rates a child or the more anxious a child rates themselves, the more likely they are to rate themselves as highly symptomatic across all scales of the corresponding ASEBA instruments. Dimensional extremes of both internalizing and externalizing symptoms may be associated with parent- and child self-reports of anxiety in ASD.

Both internalizing and externalizing symptoms occur within the ASD population, increase dysfunction, and decrease quality of life and lead to targets for intervention (39). With DSM-5, it is now possible to officially diagnose comorbid conditions in ASD, so, it is important to gain an increased understanding of the dimensional nature of other conditions present such as anxiety and ADHD (40). Further study of rating behavior of parents of children with ASD and of children with ASD is warranted and the use of the SCARED to detect anxiety in ASD should be supplemented by other measures and procedures.

In our sample, the high degree of comorbidity of ASD with other psychiatric conditions is a factor to consider. A study by Simonoff et al. found 41% of children with ASD had two or more comorbid psychiatric disorders with social anxiety (29.2%) and ADHD (28.2%) the most common (41). Our sample was a clinic-derived population from established patients from university-based clinics and diagnostic center with high levels of comorbid psychiatric diagnoses, so, our sample could be expected to have a higher degree of both internalizing and externalizing symptoms (42). The extensive psychiatric comorbidity in our sample may be a factor in the level of non-specific correlations we found between the SCARED and ASEBA instruments. Anxiety is just one of many comorbid psychiatric conditions clinicians should monitor in subjects with ASD.

Other limitations must be acknowledged in this pilot project. Our study has non-stringent clinical diagnostic procedures that limits its replicability. We used existing clinical diagnosis of ASD made by DSM-IV and DSM-5 standards and formal IQ testing was not available for all subjects with estimation of IQ based on clinical evaluation and review of available materials. DSM-5 was published in May 2013, but it was not used at our clinical sites until it gained insurance support in January 2014, so it was necessary to use DSM-IV criteria on most subjects. However, ASD is felt to be a dimensional diagnosis involving social communication, so, evaluating the use of the SCARED across the spectrum still yields clinically useful information. Future studies would improve the diagnostic reliability by using gold standard standardized assessments such as the ADOS or ADI-R and having formal intellectual assessments on all subjects. It would be helpful in future studies to further determine the impact of IQ on the degree of correlation between the SCARED and ASEBA instruments. The age range of the study was broad and not all subjects completed the YSR due to age <11 years (*N* = 71 completed the YRS). It may be necessary to follow-up with larger numbers of age 8–11 and 11–18 years to better understand developmental differences. A minority of the sample had completed TRFs for evaluation and our population only included *N* = 12 females in the sample. ASEBA surveys were all paper and pen and computer formatted entry may have increased our rate of return. There may have been a referral bias in play as subjects with ASD and symptoms of anxiety could be more likely to be referred to our study by clinicians.

The construct of emotional regulation (ER) may provide more useful ways to understand and measure the relationships between anxiety and ASD. Parent-reported “meltdowns” or “tantrums” can be understood as maladaptive involuntary forms of ER, which may be prompted by stresses in the environment (43). High-functioning patients with ASD have been shown to use involuntary forms of maladaptive ER (44). Importantly, the concept of ER difficulties in ASD provides avenues for focused and effective individualized treatment, as cognitive reappraisal techniques may be useful to help alter levels of neural activation (45).

There are likely limits to the extent any instrument relying on recall may capture signs of anxiety in ASD. Experience Sampling Methods work to capture thoughts and emotions of individuals “in the moment” and may help identify the stresses and contexts of daily life experiences that can lead to involuntary maladaptive forms of ER interpreted as anxiety. This method has been found to help understand the nature and quality of social experiences in children with ASD and holds promise in improving the accuracy of self-report in this population (46, 47).

The SCARED showed utility in assessing signs and symptoms of anxiety in high-functioning ASD and converged with the CBCL scales measuring anxiety. Also elevated scores on the CBCL syndrome scale for anxious/depressed predicted both child and parent scores of anxiety on the SCARED. The results suggest that children with ASD can self-report symptoms of anxiety. Parents report multiple behavioral and emotional concerns in their children with high levels of anxiety while youth with ASD may more likely interpret internalized symptoms as anxiety. Reasonable agreement between parent and child SCARED ratings of anxiety in ASD were shown. However, there is a great degree of non-specific correlations between parent and child ratings of anxiety on the SCARED and ASEBA scales.

Both instruments contribute to an understanding of the dimensional nature of anxiety in ASD. The SCARED can be utilized in children with higher functioning ASD to screen for anxiety symptoms. Since anxiety in ASD can respond to treatment, it is helpful to target symptomatic areas identified by the SCARED through evidence-based treatments such as CBT. However, the use of the SCARED in children with ASD should be supplemented by further multimodal evaluation and rating instruments.

## Ethics Statement

All procedures performed in this study involving human participants were in accordance with the ethical standards of the University of Louisville and with the 1964 Helsinki declaration and its later amendments or comparable ethical standards.

## Author Contributions

WL, KD, PW, GK, and LS planned and carried out study. TW and RK assisted with study design and statistical analyses. WL obtained grant funding for project through intramural research program.

## Conflict of Interest Statement

The authors report no conflicts of interest with the conduct of this study or publication of this manuscript. The funding of the study from the University of Louisville School of Medicine Department of Pediatrics Pilot Research Grant Program did not lead to any conflicts of interest regarding the publication of this manuscript.
